# Intestinal helminth infections amongst HIV-infected adults in Mthatha General Hospital, South Africa

**DOI:** 10.4102/phcfm.v7i1.910

**Published:** 2015-12-09

**Authors:** Olukayode A. Adeleke, Parimalaranie Yogeswaran, Graham Wright

**Affiliations:** 1 Department of Family Medicine, Walter Sisulu University, Mthatha, South Africa; 2 Department of Family Medicine and Rural Health, Walter Sisulu University, Mthatha, South Africa; 3 Centre for Health Informatics Research and Development, University of Fort Hare, South Africa

## Abstract

**Background:**

In South Africa, studies on the prevalence of intestinal helminth co-infection amongst HIV-infected patients as well as possible interactions between these two infections are limited.

**Aim:**

To investigate the prevalence of intestinal helminth infestation amongst adults living with HIV or AIDS at Mthatha General Hospital.

**Setting:**

Study participants were recruited at the outpatient department of Mthatha General Hospital, Mthatha, South Africa.

**Methods:**

This cross-sectional study was conducted between October and December 2013 amongst consecutive consenting HIV-positive adult patients. Socio-demographic and clinical information were obtained using data collection forms and structured interviews. Stool samples were collected to investigate the presence of helminths whilst blood samples were obtained for the measurement of CD4+ T-cell count and viral load.

**Results:**

Data were obtained on 231 participants, with a mean age of 34.9 years, a mean CD4 count of 348 cells/µL and a mean viral load of 4.8 log_10_ copies/mL. Intestinal helminth prevalence was 24.7%, with *Ascaris Lumbricoides* (42.1%) the most prevalent identified species. Statistically significant association was found between CD4 count of less than 200 cells/µL and helminth infection (*p* = 0.05). No statistically significant association was found between intestinal helminth infection and the mean CD4 count (*p* = 0.79) or the mean viral load (*p* = 0.98).

**Conclusion:**

A high prevalence of intestinal helminth infections was observed amongst the study population. Therefore, screening and treatment of helminths should be considered as part of the management of HIV and AIDS in primary health care.

## Abstrait

**Les infections par les helminthes intestinaux chez les adultes séropositifs à l'Hôpital Général de Mthatha, en Afrique du Sud**.

**Contexte:**

Peu d’études ont été faites en Afrique du Sud sur la prévalence de la coïnfection par les helminthes intestinaux chez les patients séropositifs ainsi que les interactions possibles entre ces deux infections.

**Objectif:**

Etudier la prévalence d'infection par les helminthes intestinaux chez les adultes séropositifs avec le VIH ou le SIDA à l'Hôpital Général de Mthatha.

**Lieu:**

Les participants à l’étude ont été recrutés au service de consultations externes de l'Hôpital Général de Mthatha, à Mthatha en Afrique du Sud.

**Méthodes:**

Cette étude transversale a été faite d'octobre à décembre 2013 chez des patients séropositifs consécutifs, adultes et consentants. On a obtenu les informations sociodémographiques et cliniques au moyen de formulaires de collecte de données et d'entretiens structurés. Des échantillons de selles ont été prélevés pour déterminer la présence d'helminthes et des échantillons sanguins ont été obtenus pour mesurer le nombre de cellules T CD4+ et la charge virale.

**Résultats:**

On a obtenu des données de 231 participants, avec un âge moyen de 34.9 ans, une moyenne de cellules CD4 de 348 cellules/µL et une charge virale moyenne de 4.8 log10 copies/mL. La prévalence d'helminthes intestinaux était de 24.7%, avec les *Ascaris Lumbricoides* (42.1%) qui étaient les espèces les plus fréquentes. On a trouvé un lien important du point de vue statistique entre le nombre de CD4 de moins de 200 cellules/µL et l'infection par les helminthes (*p* = 0.05). Aucune corrélation importante du point de vue statistique n'a été trouvée entre l'infection par les helminthes intestinaux et le nombre moyen de CD4 (*p* = 0.79) ou la charge virale moyenne (*p* = 0.98).

**Conclusion:**

On a remarqué une forte prévalence d'infections par les helminthes intestinaux chez les sujets à l’étude. Ainsi, le dépistage et le traitement des helminthes devrait faire partie de la gestion du VIH et du SIDA dans les soins de santé primaire.

## Introduction

Helminths are the most common parasitic agents of humans in Africa and other developing countries, producing a burden of disease that exceeds better-known conditions, including malaria and tuberculosis.^1^ Sub-Saharan Africa bears an inordinate proportion of the global HIV pandemic, being home to more than two-thirds of the estimated 35.3 million people worldwide living with HIV in 2012.^2^ There is significant overlaps in the geographical distribution of HIV and helminthiasis, and co-infection is highly endemic in this region.^3^

South Africa has the highest HIV epidemic in the world,^2^ with an estimated 10% of the total population, approximately 5.2 million people, living with HIV or AIDS in 2013.^4^ A significant proportion of South Africans live in areas characterised by poverty and under-development,^5^ conditions that have been shown to fuel the continuing spread of HIV and helminthic infections.^6^ Although the national estimates of helminth prevalence are not known, data from surveys in various parts of South Africa reveal infestation levels that range between 70% and 100% in school-age children and pre-schoolers.^5^ For example, the prevalence of intestinal parasites amongst school children in four schools in Mthatha is reported to be 64.8%.^7^ In addition, helminths were identified in 21.7% of stool specimen from adults obtained from some public hospital laboratories in Kwazulu-Natal Province.^8^ However, data on the prevalence of intestinal parasites in the country's adult population are insufficient.

Despite the high prevalence of these two infections in Sub-Saharan Africa, the effect of intestinal helminths on the epidemiology of HIV infection, including the risk of HIV transmission and disease progression and management, remains uncertain.^9^ Studies have shown that infestation by intestinal worms adversely affects the immune response, causing immune dysfunction and diminishing the host's capacity to mount appropriate defence against infections such as tuberculosis and HIV infection.^10,11^ Although they vary in their biology, route of infection and developmental stages in which they exist in the human host, most helminth parasites evoke a similar immune response in the host. This entails a polarisation of CD4+ T-cells towards a Th2 phenotype, with reduced Th1-type responses, as well as immunosuppression of both worm-speci?c and generalised immune responses.^12^ Th1 cells are important in eliminating intracellular pathogens, and inflammatory responses. Th2 cells, on the other hand, target extracellular pathogens and produce cytokines, which enhance antibody production. The profound Th2 and diminished Th1 responses as well as the chronic immune activation found in patients with intestinal helminth infection have been hypothesised to lead to an increased susceptibility to HIV infection and enhanced HIV replication in helminth-infected individuals.^12,13^

Intestinal helminthic infection has been shown to be common amongst people living with HIV or AIDS in Africa, with prevalence ranging from 17% to 66% depending on the methods used to identify the helminths.^5,9,14^ However, studies conducted in several African countries have produced conflicting results regarding the impact of intestinal helminthic infection on the epidemiology of HIV. Though most authors agree on the potential theoretical deleterious immunological consequences of HIV and helminthic infection co-existing in an individual, the results from various studies evaluating the practical implication of treating intestinal helminthic infection in HIV disease progression are inconclusive.^15^ Whilst some studies found no effect of treatment of intestinal helminth infections on HIV disease progression,^9,16,17^ other authors have reported a beneficial effect, with a reduction in HIV viral load, including reduction in the risk of HIV transmission following treatment of intestinal helminthic infection.^18,19,20^

This study sought to explore the extent of the problem of intestinal infestation in HIV-positive adults at the Mthatha General Hospital, a district hospital located in the OR Tambo district of the Eastern Cape Province, South Africa. About 12.6% of the South African population lives in the province, the third largest and one of the poorest in the country, with the highest percentage of households living without toilet facilities.^21^ Although there is a paucity of data on the prevalence of HIV-helminth co-infection in the province, the endemic poverty, lack of access to clean water and poor sanitation makes it highly probable that HIV-helminth co-infection will be common in this area.

## Research methods and design

### Study design, setting and sampling strategy

This cross-sectional study was conducted within the outpatient department of Mthatha General Hospital, Mthatha, Eastern Cape Province of South Africa. This is a district hospital that is a part of the Mthatha Academic Hospital Complex, one of the teaching complexes of the Faculty of Health Sciences, Walter Sisulu University. Consecutive patients aged 18 years and above, confirmed to be HIV-positive and HAART (Highly active antiretroviral therapy)-naïve, were enrolled between October and December 2013. They were provided with written and verbal information about the study and were required to provide written informed consent. Exclusion criteria were the presence of acute severe illnesses like pneumonia, tuberculosis or meningitis. Over a three-month period, the population of HAART-naïve HIV-positive adults attending the outpatient department was estimated at 720. As the prevalence of helminth infection amongst the population was unknown, this was computed as 50%. Using a confidence level of 95% and a design effect of 1.0, a sample size of 251 was calculated using OpenEpi software.^22^ None of the eligible participants declined to participate in the study.

### Collection, transport and processing of specimens

Stool samples were collected according to World Health Organization (WHO) standard procedure.^23^ Submitted stool specimens were transported in a cooler box to the department of Medical Microbiology at Walter Sisulu University within an hour of collection for microscopy. About 5 mL of venous blood specimen was collected in ethylenediaminetetraacetic acid (EDTA) and plasma preparation tubes, for estimation of CD4 count and plasma HIV-RNA level, immediately after collection. A pilot study was conducted amongst 20 participants who were not included in the main study. Necessary adjustments were made to the interview guide and specimen collection technique. For example, all 20 participants strongly objected to the application of transparent adhesive tape to skin around the anus to obtain ova of *Enterobius vermicularis* (the scotch-tape technique). The technique was omitted in the main study.

Stool specimens were processed using the formalin-ethyl acetate concentration method. An experienced medical technologist performed microscopy of the concentrated specimen, and a consultant medical microbiologist examined a random selection of the microscope slides for the purpose of quality control. Parasites were quantified based on the number of parasites seen per high microscopic power field (hpf). CD4 count was determined at the National Health Laboratory Service in Mthatha from venous blood using the Panleucogating (PLG) methodology, whilst HIV viral load was determined using COBAS^®^ AmpliPrep/COBAS^®^ TaqMan^®^ HIV-1 Test, version 2.0.

Participants identified as having intestinal helminth infection were treated with anti-helminthic drugs according to standard practice. Participants who were eligible for antiretroviral therapy based on CD4 count were contacted and appropriately referred for treatment initiation.

### Statistical analysis

Data analysis was performed using the Statistical Package for the Social Sciences (SPSS) version 21.0 (SPSS Inc., Chicago, IL, USA). Data were expressed as means ± standard error (s.e.) of the mean or as means ± standard deviation (s.d.) for the continuous variables and proportions (percentages) for the categorical variables. The chi-square test or Fischer's exact test was used to test the degree of association of categorical variables. Student's *t*-test was performed to assess differences between two means and analysis of variance (ANOVA) between groups. All tests were two-sided and a *p*-value of ≤ 0.05 was considered significant.

## Ethical considerations

Ethical approval was obtained from the Human Research Committee of Walter Sisulu University (Protocol number: 005/2013). Institutional approval to conduct the research was obtained from the Eastern Cape Department of Health, Bisho.

## Results

A total of 252 HIV seropositive adults were enrolled in the study. Twenty-one of these (6 male, 15 female) were excluded from the analysis because of incomplete data. The excluded participants were not different from the remaining participants in terms of age, gender, level of education, income, employment status, CD4 count or viral load. Of the remaining 231 HIV seropositive patients with complete data, 72 (31%) were male and 231 (69%) female. The mean age of the study participants was 34.9 years (s.d. = 10.6 years), ranging from 18 years to 64 years. Other social, demographic and clinical characteristics of the participants are presented in Table 1.

**TABLE 1:** Socio-demographic characteristics of the study population (*n* = 231).

Characteristics	Total participants (*n*= 231) *n*(%)		Helminth-infected (*n*= 57) *n*(%)		Helminth-uninfected (*n*= 174) *n*(%)		ANOVA (*p*-value)
Age (years), mean ± s.d.	34.7 ± 10.6		35.4 ± 9.7		34.7 ± 10.9		0.66
**Gender**							**0.36**
Male	72	31.2	15	26.3	57	32.8	0.41
Female	159	68.8	42	73.7	117	67.2	0.40
Number of household members, mean ± s.d.	5 ± 4		6 ± 7		5 ± 3		0.05
**Employment**							**0.09**
Yes	99	42.9	31	54.4	68	39.1	0.09
No	132	57.1	26	45.6	106	60.9	0.09
**Level of education**							**0.5**
None	4	1.7	2	3.5	2	1.2	0.23*
Primary	43	18.6	10	17.5	33	18.9	0.81*
Secondary	146	63.2	38	66.7	108	62.4	0.53*
Tertiary	38	16.5	7	12.3	31	17.9	0.32*
**Average monthly income (Rand)**							**0.51**
< 1000	125	53.7	27	47.4	98	56.3	0.27*
1000 to 5000	86	37.5	25	43.8	61	35.1	0.26*
> 5000	20	8.7	5	8.8	15	8.6	0.96*

ANOVA, analysis of variance (calculated with Asymp. Sig 2-sided or Exact Sig 2-sided ANOVA); s.d., standard deviation; *, 2-tailed *Z* -score test

Of the 231 participants, 132 (57.1%) were unemployed, the majority of whom were dependent on various forms of social grants. The average monthly income of 53.7% of these patients was less than R1000. There was no statistically significant association between participants’ age, gender, level of education or average monthly income and intestinal helminth infection. Helminth-infected participants reported a higher number of household members (mean = 6) than their helminth-uninfected counterpart (mean = 5) (*p* = 0.05).

As shown in Table 2, 163 (70.6%) participants had access to tap water, whilst 178 (77.1%) used pit latrines, mostly shared with other families. There was no statistically significant association between source of water or toilet type and helminth infection. Only 6.1% of the participants indicated that they clean their hands with soap and water after defecation, and 93.1% indicated that they wash their hands with water only. No statistically significant association was found between hand-washing practices and intestinal helminthic infection (*p* = 0.076).

**TABLE 2:** Sanitation-related characteristics of the study population (*n* = 231).

Characteristics	Total participants(*n*= 231) *n*(%)		Helminth-infected (*n*= 57) *n*(%)		Helminth-uninfected (*n*= 174) *n*(%)		ANOVA (*p*-value)
**Water source**							**0.37**
Tap	163	70.6	39	68.4	124	71.3	0.68*
River	48	20.8	12	21.1	36	20.7	0.95*
Tank	20	8.7	6	10.6	14	8	0.56*
**Toilet type**							**0.45**
Pit	178	77.1	46	80.7	132	75.9	0.59
Flush	53	22.9	11	19.3	42	24.1	0.59
Bucket	0	0	0	0	0	0	
**Refuse disposal**							**0.76**
Burning	172	74.5	44	77.2	128	73.6	0.58*
Municipal collection	58	25.1	13	22.8	45	25.8	0.64*
Others	1	0.4	0	0	1	0.6	0.56*
**Hand care**							**0.20**
None	2	0.9	0	0	2	1.1	0.41*
Water and soap	14	6.1	1	1.8	13	7.5	0.11*
Water only	215	93.1	56	98.2	159	91.4	0.11*

ANOVA, analysis of variance (calculated with Asymp. Sig 2-sided or Exact Sig 2-sided ANOVA); *, 2-tailed *Z*-score test

**FIGURE 1:**
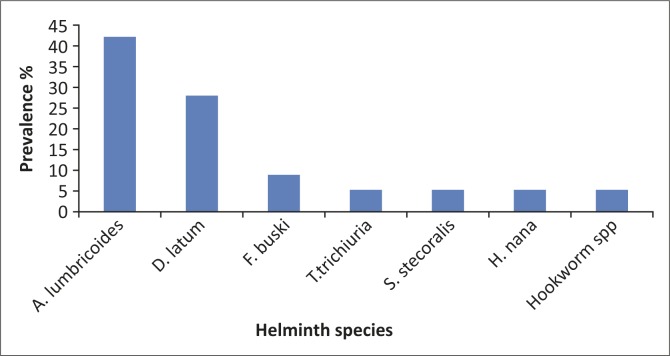
Prevalence of helminth species amongst helminth-infected participants (*n* = 57).

Of the 231 participants whose stools were examined for intestinal parasites, helminth infection was detected in 57, representing 24.7% of the study population. Seven species of helminths were identified: *Ascaris Lumbricoides* (42.1%), *Trichuris trichura* (5.26%), *Strongyloides stercoralis* (5.26%), Hookworm species (5.26%) (*Necator americanus, Ancylostoma duodenale*), Hymenolepis *nana* (5.26%), *Diphylobothrium latum* (28.07%) and *Fasciolopsis buski* (8.8%) (Figure 1).

The CD4+ cell count of the study participants ranged widely from 3 to 1283 cells/µL, with a mean of 348 cells cells/µL. Sixty-nine participants (29.9%) were severely immunosuppressed with a CD4+ cell count of less than 200 cells/µL, of whom 37 (16%) had a CD4 count of less than 100. A total of 105 (45.4%) patients had a CD4+ count of more than 350 cells/µL. The mean plasma viral load for the study participants was 4.8 log_10_ copies/mL (Table 3). Although the participants with helminth infection had a slightly lower mean CD4 count and a slightly higher mean viral load than those with no infection, there was no statistically significant differences in the mean viral load (*p* = 0.98) or mean CD4+ cell count (*p* = 0.79) in both groups. However, low CD4 count (< 200 cells/µL) was associated with intestinal helminthic infection. This was statistically significant (*p* = 0.05).

**TABLE 3:** CD4+ T-cell count and HIV viral load of the study population.

CD4+ cell count and viral load	Total participants (*n*= 231) *n*(%)		Helminth-infected (*n*= 57) *n*(%)		Helminth-uninfected (*n*= 174) *n*(%)		ANOVA (*p*-value)
**CD4+ cell count category**							**0.14**
< 200 cells/µL	69	29.9	23	40.4	46	26.4	0.05*
200–350 cells/µL	57	24.7	12	21.0	45	25.9	0.47*
350 cells/µL	105	45.4	22	38.6	83	47.7	0.23*
**Log10 HIV-RNA category (copies/mL)**							**0.35**
≤ 3 log10	11	4.8	1	1.8	10	5.7	-
3–5 log10	120	51.9	33	57.9	87	50	-
5 log10	100	43.3	23	40.4	77	44.3	-
CD4+ cell count, mean (±s.d.) cells/µL	348 ± 239		340 ± 264		350 ± 232		0.79
Log10 HIV-RNA, mean (±SEM) copies/mL	4.8 ± 0.06		4.8 ± 0.08		4.7 ± 0.11		0.98

ANOVA, analysis of variance (calculated with Asymp. Sig 2-sided or Exact Sig 2-sided ANOVA).

*, 2-tailed *Z*-score test; s.d., standard deviation; SEM, standard error of the mean; log, logarithm.

Apart from the seven species of intestinal helminth identified during the study, *Entamoeba histolytica*, a protozoan, was identified in two of the participants (0.9% prevalence) whilst the bacterium *Escherica coli* was found in 35 patients (15.2% prevalence). Thirteen (5.5%) of the participants had mixed infection (Table 4).

**TABLE 4:** Multiple infections amongst the study participants (*n* = 231).

Parasites	Prevalence (*n*)	Prevalence (%)
*A. lumbricoides* and *E. coli*	4	1.73
*A. lumbricoides* and *F. buski*	1	0.43
*A. lumbricoides*and *E. histolytica*	1	0.43
*E. coli* and *H. nana*	5	2.2
*E. coli* and *D. latum*	1	0.43
*E. coli* and *F. buski*	1	0.43

## Discussion

Nearly 70% of participants in the study were female (Table 1), which might be an indication of low attendance of men at the outpatient department of the hospital. It has been noted that in most part of Africa, men in general seek treatment for all ailments (HIV/AIDS-related or otherwise) less often than women. This has been attributed to the culture of ‘tough’ masculinity which assumes that men are more powerful and less vulnerable than women.^24^ It has also been shown that more women than men accessed HIV and/or AIDS care and treatment^25^ and were more than twice as likely to be on anti-retroviral therapy (ART) than men.^26^

The study found that 178 (77.1%) of the participants use pit latrines. This is similar to findings from a community-based survey conducted in 2008 by Mfenyana et al.^27^ in which only 17.9% of the households had access to a flush toilet. The low socio-economic status noted amongst the participants in this study could be because of the setting of the study. As noted by Nattrass,^24^ people of higher socio-economic status are less likely to use public health facilities than those of lower socio-economic status. Consistent with the findings of Modjarrad et al.^28^ as well as Mwambete et al.^29^ the participants’ age, gender, income, level of education or occupation were not predictive of helminth status in the present study.

A statistically significant association was found between a large number of household members and helminthic infection amongst the participants. This was similar to the findings of the report by Anuar et al.^30^ in which increased number of household members was associated with a higher frequency of intestinal parasitic infections. Crowded living conditions in developing countries have been shown to be one of the factors that increase susceptibility to soil-transmitted helminth infection.^31^

Although no statistically significant association was found between hand washing and helminthic infection, the proportion of participants with helminth infection who used soap and water was smaller than those without intestinal helminth infection (1.8% vs. 7.5%; *p* = 0.11). The lack of a statistically significant association in this study could be as a result of the small sample size. A review by Strunz et al.^32^ shows that hand washing with soap after defecation significantly reduces the risk of helminth infection. Primary care physicians and indeed all health care workers practising in this community should therefore opportunistically provide patients with information on hand-washing practices and motivate a behavioural change during every consultation.

The species of intestinal helminths detected in this study and their prevalence were similar to the findings of some of the previously published reports of helminth infection amongst adults living with HIV and AIDS in Africa.^8,28,33^ However, the 24.7% prevalence found in the present study was significantly lower than those from several other studies, which range from 48.7% to 66%.^5,34^ This difference in prevalence reported in the various studies could be because of the differences in sample sizes, setting of the studies, inclusion and exclusion criteria used in participant selection as well as the difference in the methods used in the identification of the parasites (stool microscopy with or without serology)^5^.

The Mthatha region of the Eastern Cape Province of South Africa has one of the highest prevalences of *Taenia solium* neurocysticercosis in the country.^35^ The absence of the eggs of tapeworms in any of the stool specimens examined is therefore noteworthy, considering the high prevalence of neurocysticercosis in the community. The absence could be attributed to the small sample size or could be a result of HIV enteropathy, which affects the colonisation of the intestinal tract by parasites.^36^ Larger studies should be conducted to explore the relationship between the prevalence of *T*. *solium* and HIV infection in this community.

The mean CD4 count of the study population was 348 cells/µL. The relatively lower CD4 count of the participants in this study could be attributed to the fact that this was a facility-based study, involving individuals who are more immunosuppressed than those found in the community. In this study, 69 (29.9%) of the participants were considerably immunosuppressed with a CD4 count of less than 200.

Consistent with the findings of Mwambete et al.^29^ a statistically significant association was observed in the present study between a CD4 count less than 200 cells/µL and helminth infection (Table 3). This is in contrast to the report by Walson et al.^14^ who noted an increased prevalence of intestinal helminth infection amongst patients with higher CD4 counts. The correlation of helminth infection with higher CD4 count found by Walson et al.^14^ could be attributed to the inclusion in the study of only individuals with CD4 counts greater than 250 cells/µL and without WHO Stage 3 or 4 disease. This represents patients with less severe immune suppression than those in the present study.

No statistically significant difference was found between the mean CD4+ cell count (*p* = 0.79) of the helminth co-infected participants compared to the helminth-uninfected. This is similar to findings from similar studies conducted in Uganda,^17^ South Africa^5^ and Ethiopia.^37^ In addition, no statistically significant association was observed between intestinal helminth infection and viral load in this study (*p* = 0.98), similar to the findings of Modjarrad et al.^38^ and Hosseinipour et al.^9^ but contrary to the findings of others.^5,37^

The lack of association between CD4 count, viral load and intestinal helminth infection in the present study could be attributed to the small sample size as well as possible under-estimation of the presence of intestinal helminth because stool microscopy was used as the sole method of diagnosing helminthiasis. It could also be attributed to the difference in the prevalence of the various helminth species identified in this study compared to those identified in studies where association has been found between CD4 count or viral load and helminthic infection. As Mkhize-Kwitshana et al.^5^ have argued, diagnosis of helminthiasis based on stool microscopy alone could lead to a wrong classification of infection or non-infection and could affect the evaluation of the immunological effect of helminth co-infection on HIV and AIDS. Further studies that incorporate monitoring of markers of immune activation are necessary in order to provide a better understanding of the HIV-helminth relationship.

## Limitations

As a cross-sectional survey, this study is useful in generating hypothesis but cannot show any causal or risk relationships. The use of a hospital-based population in this study was not representative of the adult population in Mthatha. Moreover, the study population may not be representative of all patients at the outpatient department because of the use of convenient sampling.

HIV-negative individuals were not included in this study to serve as a comparison group. As a result of this, comparative inferences cannot be made on the prevalence of helminth infections across populations of differing HIV status. Only one stool specimen was collected from each participant and helminth detection was based only on the use of light microscopy. These could have led to an under-estimation of the prevalence of helminthiasis.

## Recommendation

It is recommended that larger community-based studies be conducted to determine the extent of intestinal helminth co-infection amongst people living with HIV or AIDS. Future studies should include HIV-negative comparison groups and be appropriately designed to explore several aspects of HIV-helminth interactions, including the effect of the various helminth species as well as multiple infections on HIV disease. Diagnosis of intestinal helminth infections in such studies should include, in addition to microscopy, parasite serology and molecular diagnostic techniques.

## Conclusion

The study revealed that intestinal helminth infection was relatively common amongst the study population, with 24.7% prevalence. Helminthiasis was diagnosed in this study using a simple and cost-effective technique of stool microscopy, but the prevalence of intestinal helminth could actually be higher if other methods of identification (such as serology) were combined with microscopy.

Intestinal helminthic infections are known to be associated with high morbidity, adversely affecting the host's general health and nutritional status.^39^ In HIV-positive patients, this represents an additional burden on already compromised hosts. In this study, no statistically significant association was found between intestinal helminth infection and markers of HIV disease progression; however, intestinal helminth infection was found to be significantly associated with lower CD4 count (< 200 cells/µL). Routine anti-helminth treatment should therefore be included as part of patients’ comprehensive care, at least amongst this group of patients.
